# Effect of polystyrene nanoplastics on its toxicity and reproduction in *Philodina roseola*

**DOI:** 10.1038/s41598-025-98637-1

**Published:** 2025-04-23

**Authors:** B. Praveen Kumar, Sujithra Vijayakumar, John Thomas

**Affiliations:** Centre for Nanobiotechnology, Vellore Institute of Technology (VIT), Vellore, Tamilnadu 632014 India

**Keywords:** Nano polystyrene, Rotifers, Toxicity, Antioxidants, Reproduction, Ecology, Environmental sciences

## Abstract

Micro-nano plastics have emerged as a major ecological concern. The nanoplastics (NPs) pose a huge threat to microscopic animals. Our study aims to decipher the effect of polystyrene nanoplastics (PSNPs) of 50 and 100 nm sizes on a bdelloid rotifer (*Philodina roseola*). Both sizes of PSNPs were analyzed using field emission Scanning electron microscopy, Fourier transform Infrared spectroscopy, and Dynamic light scattering analyses. The LC_50_ values for 50 and 100 nm PSNPs at 48 h upon interaction with the rotifers were 16.36 and 22.94 mg/L respectively. The total protein and superoxide dismutase levels decreased with an increase in concentration in both PSNPs upon interaction at various concentrations (4, 8, 12, and 16 mg/L). Whereas the lipid peroxidase and reactive oxygen species levels increased with an increase in concentration for both PSNPs at similar concentrations. Further, both PSNPs were found to cause internal organ damage in rotifers. A delay in the hatching rate was observed when the rotifers interacted with both PSNPs in addition to the decrease in the hatching rate of F_1_ generation. Therefore, PSNPs pose a threat to the natural life cycle in the rotifers.

## Introduction

Global plastic production has been increasing over the last few decades and at present accounts for approximately 300 million tons per annum^1^. Generally, plastics are discarded as waste after being used. The increasing population, waste mismanagement issues, industrial developments, and increasing plastic waste disposal led to the accumulation of plastics in the environment causing serious concern. These plastics find their way into water bodies and harm aquatic life as well as the environment. As a result, the plastics enter the food chain generating critical health issues for humans and aquatic organisms. The discarded plastics impact the surroundings based on the types of chemicals released, the type of plastic, and its size. Plastic wastes are typically solid wastes that are broadly distinguished into microplastics and macroplastics^2^.

Macroplastics (above > 5 mm in size) are observable plastics that can be seen with the naked eye. The term microplastics (MPs) comprises both “microplastics” and nanoplastics (NPs). In contrast to macroplastics, MPs belong to plastic fragments lesser than the size of 5 mm. Nanoplastics (NPs) are plastics in sizes from 2 to 200 nm formed through the degradation of MPs. To be more precise, NPs are degraded MPS with a higher size limit of 100 nm. MPs are divided into primary and secondary micro/nanoplastics (MNPs) based on their origin. Primary MNPs are less than 5 mm designed for commercial applications by industries. Whereas, secondary MNPs or “microlitter” are derived from the fragmentation or degradation of larger plastic materials. These secondary MNPs (< 5 mm) are formed as a result of the breakdown of larger plastics via physical, chemical, or biological means. These larger macroplastics found in the aquatic environments can be easily identified and removed whereas MPs and NPs become emerging environment contaminants. The size of these MPs and NPs becomes harder to detect in the aquatic environment^2–4^.

The degradation of MPs occurs more rapidly than macroplastics owing to their surface/volume ratio. Initially, it is identified visually with surface abrasions and color changes. Upon interaction with environmental factors, further degradation of macroplastics stimulates the weathering process leading to brittle plastic fragments and powdery plastic forms. The plastic degradation process is primarily governed by the physicochemical nature of the plastic polymer and various environmental factors. The molecular weight of the polymer is greatly reduced through this process and an increase in the oxygen functional groups is observed. Plastic degradation follows 6 major mechanisms that involve physical and thermal degradation processes, hydrolysis reactions, thermo-oxidative degradation, and photo/biodegradation. The physicochemical or abiotic process occurs by physical forces (waves, water currents, sediment abrasion, etc.). Thermal degradation occurs as a result of higher temperatures. The hydrolysis reactions occur due to the interaction of water with these plastics. The photo/thermo oxidation process follows a similar pattern of degradation. The biodegradation process is mediated only by the microbes, whereas the photooxidative degradation process is caused by light exposure. In general, ultraviolet (UV)-B sunlight is the prime initiator for the photodegradation reactions. The increasing exposure of these plastics to sunlight increases the photooxidation rate. In contrast, the thermo-oxidative process occurs without exposure to UV radiation followed by the catalytic reactions with oxygen. This degradation process leads to the alteration of the plastic wastes such as fragmentation into tinier plastics, surface-level damage, and discoloring of the plastics^5–7^.

NPs are usually formed through fragmentation, photooxidation, and biodegradation processes from the MPs. These accumulated NPs in the aquatic environments can be “Virgin” or may include additives or functional groups from the parent plastic material. Directly, these wastes find their way to aquatic environments through the littering process (paints, waste dumping from boats or fishing nets). Indirectly, they are accumulated from the land wastes finding routes to the aquatic environment through drainage systems, wind, or ignorance by humans. When NPs interact with the aquatic system it gets dispersed forming a colloidal solution. Further, these NPs cause aggregates with similar particles. Rivers are the main source of polluted freshwater ecosystems including the occurrence of NPs as well. Higher deposition of NPs occurs in the estuaries and rivers mainly through the aggregation process. Limited research has been conducted on the interaction of NPs with aquatic organisms^8^. Polystyrene (PS) is the most abundant type of plastic among plastic pollutants^9^. PS accounts for 6–7.8% of the total plastic production worldwide, with an annual production of over 23 million tons per year^10^.

When these plastics enter humans through the food chain, the smallest plastic in humans may cause serious issues. For instance, it can enter the lungs risking life^11^. PSNPs were found to be permeating the lipid bilayer, altering the structure of membranes and membrane proteins as well as the reduction in molecular diffusion by disturbing the overall cell functions^12^. The aquatic crustacean *Artemia salina* exposed to PSNPs showed toxicity and lethal effects on its embryonic development. Besides affecting the growth parameters, it increased the apoptosis rate highly^13^. Studies on the interaction of NPs and the aquatic invertebrate *Brachionus koreanus* further proved its toxic effects on the organism. The accumulated NPs caused oxidative stress and damage to lipid membranes^14^. *B. koreanus* exposed to zinc ions and NPs caused more toxic effects on its life cycle according to the study conducted by^15^. In similar studies, these plastics affected the lifecycle of the zooplankton *B. plicatilis* by affecting its overall lifecycle. The spawning and hatching cycle of the organism was affected greatly along with the reduced ingestion of microalgae feed^16^. Multiple studies have confirmed the ingestion of NPs by the rotifers. The abundance of NPs is greater than MPs due to the fragmentation process. These plastics and rotifers are abundantly present in both freshwater and marine environments^17^. These studies prove that NPs cause oxidative stress, damage cellular membranes, delay the hatching cycle, reproduction capacity, and mortality in micro-level aquatic organisms.

Zooplanktons like Bdelloid rotifers are ubiquitous in most aquatic habitats and are metazoans with a breadth of 100–2000 µm^18^. Rotifers are indicators of the pollution level in aquatic ecosystems^19^. They are also used as model organisms owing to their rapid sensitivity towards toxicity in aquatic systems^20^ and are considered valuable food sources for many organisms like fishes, corals, etc. As filter feeders, they are also at increased risk of ingesting MNPs. Hence, it is important to study the impact of MNPs on micro-level organisms such as rotifers. The current research work throws light on the toxic effect of polystyrene nanoplastics (PSNPs) on the freshwater rotifer, *Philodina roseola*. The freshwater rotifer *P. roseola* interacted with PSNPs of two different sizes (50 and 100 nm) and was examined to assess the toxicity of these plastics towards the test organism. The study aimed to assess the lethal concentration 50 (LC_50_) of the PSNPs and the possible toxic effects of the NPs in the hatching and reproductive ability of the rotifers. The present study uncovers the toxic impacts of PSNPs in *P. roseola* providing more understanding of plastic pollution affecting the freshwater aquatic environment of the PSNPs and the possible toxic effects of the NPs in the hatching and reproductive ability of the rotifers. It uncovers the toxic impacts of PSNPs in *P. roseola* providing more understanding of plastic pollution affecting the freshwater aquatic environment.

## Materials and methods

### Chemicals and reagents

PSNPs of sizes 50 and 100 nm were obtained from Corpuscular Inc., Cold Spring, New York, USA (Cas.no.100111-10). Dichlorofluorescein-diacetate (DCFH-DA) -2′,7 was purchased from Sigma-Aldrich. Trichloroacetic acid (TCA), thiobarbituric acid (TBA), and hydroxylamine hydrochloride were obtained from Himedia Pvt. Ltd. (Mumbai, India). Hydrogen peroxide solution (30% w/v, H_2_O_2_) and Nitro blue tetrazolium chloride (NBT) were obtained from SD Fine Chemical Ltd (Mumbai, India).

### Collection and maintenance of test organisms

**Fig. 1:**
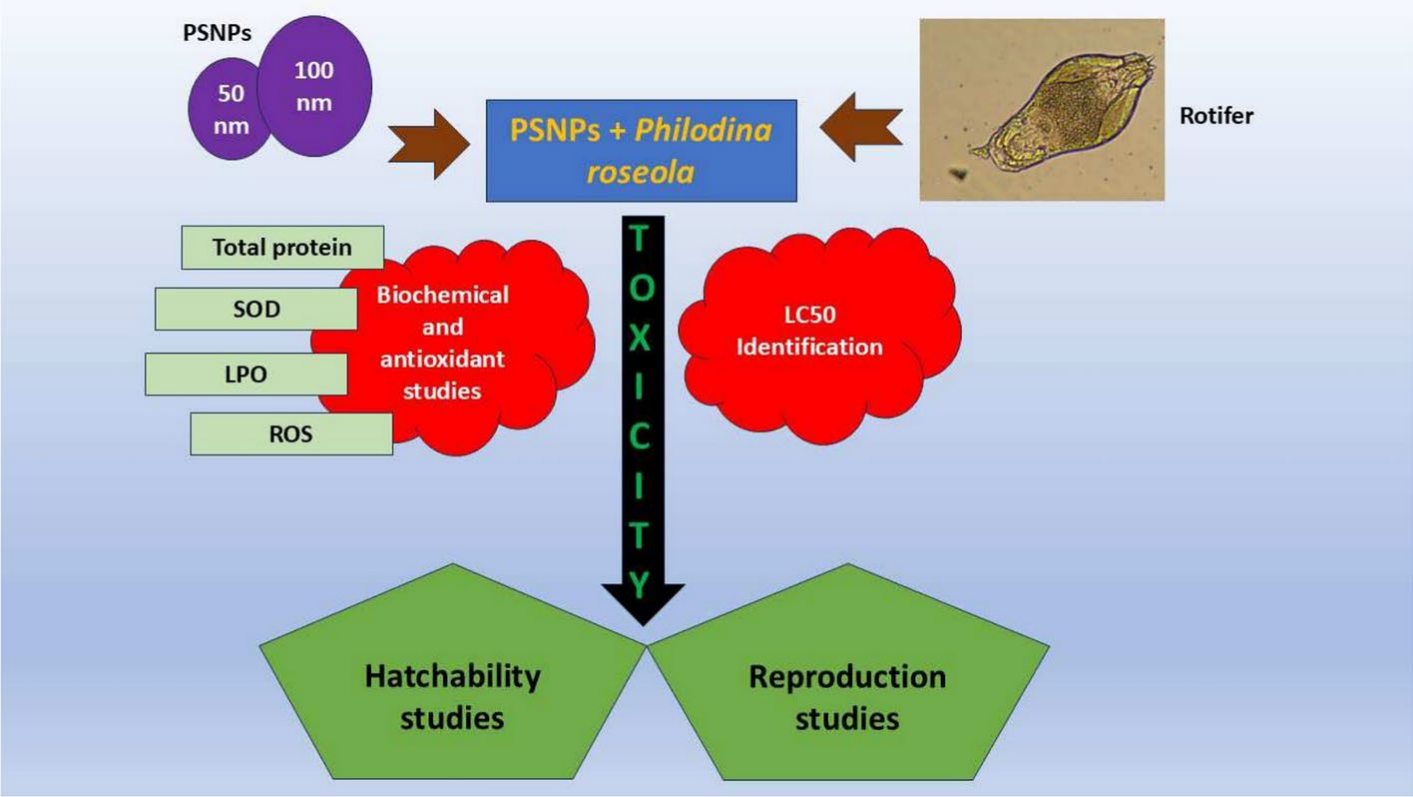
Graphical abstract of the present study.

A pictographic representation of the present study is represented as the graphical abstract in Fig. 1. The species *P. roseola falls under the* Bdelloidea class containing similar morphology. The supplementary Fig. S1 portrays the image of *P. roseola* used in the present study. The freshwater rotifer *P. roseola* (*Philodina roseola* Ehrenberg, 1832; Aphia ID: 247966; World register of marine species) was obtained from the Indian Council and Medical Research laboratory at Madurai and was maintained at 25 ± 1℃ with photoperiods of 16 to 8 h light and dark condition. The rotifer stock culture (500 mL) was added to 9.5 L of sterile freshwater and was maintained in a beaker initially for 12 days. Further, they were maintained subsequently until the end of the study when the rotifer numbers increased. The rotifers were fed with *Chlorella vulgaris* algae (freshwater *Chlorella*) twice a day on a daily basis (10^6^ cells/mL). The chlorella species were grown in BG-11 media prepared in sterile water and maintained at 20–25 °C for optimum growth. The algal stock cultures were maintained regularly in the laboratory until the experiment was done. *Chlorella* species are rich in protein and are used as feed for rotifers^21,22^.

### Characterization of PSNPs

The surface morphology of the different sizes of PSNPs was characterized by field emission scanning electron microscopy (FE-SEM) (EV018CarlZeiss, Germany). NPs were characterized through Fourier transform infrared spectroscopy (FTIR) and Dynamic light scattering (DLS) analysis to analyze the effect of chemical bonding and electrostatic force similar to the methodology followed by^23^ with slight modifications. The PSNPs were subjected to FTIR and DLS before interaction with *P. roseola*. The FTIR and DLS analysis are explained in detail as follows.

#### FTIR spectroscopy of PSNPs

FTIR spectroscopy, [FT/IR 6800 type A from Jasco, Japan] was used for the characterization of the NPs. FTIR spectroscopy analyses the changes in the entire composition of biomolecules by resolving modifications of functional groups^24^. All the FTIR spectrophotometry of samples was performed by transmittance method using the Potassium bromide (KBr) disc method. The PSNPs stock suspension was made with filtered deionized water (1 mg/mL—both sizes 50 and 100 nm). The suspension was further sonicated for 15–30 min for effective dispersion in filtered MilliQ water. The NPs of both sizes used for the interaction study with the rotifers were analyzed to study the peak shifts caused by plastic leaching in the time period of 0 hand 72 h. The transmittance peaks from 4000 to 400 cm^−1^ were analyzed post baseline corrections.

#### DLS analysis of PSNPs

The light scattering technique is used to characterize the dispersion behavior of particles in mixtures developed from a series of fundamental identifications^25^. The Z-average of the dispersed NPs was quantified using a nanoparticle analyzer (SZ100, Horiba Scientific, Japan). A stock suspension of NPs made in sterile water (25,000 mg/L) was used to prepare different test concentrations (10, 20, 30, 40, and 50 mg/L) of the NPs with filtered fresh water. The size of the NPs was observed using DLS from 24 to 72 h. The data was assessed in triplicates and presented as mean ± S.D.

### Acute toxicity assessment of the PSNPs in *Philodina roseola*

#### Experimental design

The experimental setup for the acute toxicity studies was performed using the previously reported protocols by^26^ and^27^ with certain modifications. The acute toxicity experiments were conducted in adult rotifers (male and female rotifers) that were grown from the initial neonate stage from the stock culture. In addition, the eggs from the grown adults were collected for further studies. An equal number of animals belonging to both sexes was taken for assessing the toxicity study. The toxicity of PSNPs was determined by following the method described in^28^. The toxicity of 50 and 100 nm NPs was studied in adult rotifers at different concentrations (10, 20, 30, 40, and 50 mg/L). The experiments were conducted in 24 well plate containing 10 rotifers (both male and female; n = 10) in each well. All the experiments were carried out in triplicates. The animals were monitored daily for mortality up to 72 h. Additionally, the percentage of mortality was calculated. LC_50_ values were assessed using probit analysis. Adult rotifers that were not treated with NPs served as controls. After the study period, TEM analysis was used to identify the presence of PSNPs within the test organisms. The ingestion of PSNPs and other morphological changes were observed. Damage to the morphology of *P. roseola* was also studied under 40X magnification. Vertical sections of *P. roseola* were characterized for the presence of PSNPs by TEM. Prior to TEM analysis, the samples were preserved on a glass slide. The treated and control rotifers were homogenized and dried by lyophilization for 12 h to remove the water content in the samples.

#### Biochemical parameters evaluation assay

In the scenario of aquaculture, evaluation of the oxidative range of aquatic organisms is important as it directly impacts their physical and metabolic state harming their nutritional values. Oxidative stress or damage in the macromolecules (Lipid/Protein/Deoxyribonucleic acid) of the organisms occurs as a result of an imbalance between antioxidant production and defense mechanisms. Rotifers are commonly used in ecotoxicological studies owing to their ecological importance and also as live feeds in the aquaculture sector. Various studies support the assessment of Reactive oxygen species (ROS) and lipid peroxidation (LPO) in rotifers as it is directly connected to the aerobic metabolism and food consumption of the organism. Superoxide dismutase (SOD) is analyzed as a significant eco-toxicity biomarker in numerous aquatic beings using its enzyme activity. Earlier research on rotifers such as *B. plicatilis* and *B. calyciflorus* cloned the Mn-SOD gene for the study of its impact on oxidative stress through mRNA expression. These studies highlight the importance of SOD enzyme in the role of aging and toxicity. The food intake and the nutritional quality of the rotifers are interrelated and it is essential to identify the protein levels^29–31^.

Based on the LC_50_ values obtained by the toxicity studies, the concentration for biochemical assays was determined. The biochemical parameters were evaluated by following the protocols of^32^. The total protein, SOD, LPO, and ROS levels were estimated for the rotifers. Adult rotifers were treated with 4, 8, 12, and 16 mg/L concentrations of 50 nm and 100 nm PSNPs. The treated animals were used for the biochemical analysis. Adult rotifers that were not treated with NPs served as controls.

##### Total protein quantification

Total protein content was measured in the control and treated rotifer homogenates according to the Bradford method^32,33^. To quantify the protein concentration, bovine serum albumin was used as a standard. Control and treated rotifers were homogenized with 500 µL 1 × Phosphate buffered saline and centrifuged at 7000 rpm for 10 min. The supernatant was collected and used for the biochemical assays. 10 µL of Bradford reagent, methanol, and 85% Phosphoric acid were added to 100 µL of the supernatant. The samples were then incubated in the dark for 30 min. The absorbance was recorded at 595 nm using an UV–Visible (UV–vis) spectrophotometer.

##### SOD level quantification

SOD activity was determined using the method described by^20,32^. In this assay, NBT was mitigated by hydroxylamine in the presence of EDTA and the reduction was measured at 560 nm. 100 μL of homogenate supernatant was mixed with 20 mM hydroxylamine hydrochloride (10 μL), 96 mM NBT (10 μL), 50 m M Sodium carbonate (130 μL), and 0.6% TritonX-100 (10 μL). The mixture was then incubated at 37 °C for 20 min and absorbance was measured at 560 nm.

##### LPO activity quantification

The total amount of malondialdehyde (MDA) produced by the organisms was estimated using a lipid peroxidation assay as per standard protocol. 50 µL of homogenate supernatant was mixed with 2 mL of 0.25% (w/v) TBA in 10% w/v TCA. The obtained samples were heated at 90℃ for 30 min, then flash cooled on ice for 5 min and centrifuged for 10 min at 7000 rpm. The absorbance of the supernatant was measured from 532 to 600 nm using a UV–vis spectrophotometer (Model U2910, HITACHI, Japan). MDA concentration was determined using a standard graph^32,34^.

##### ROS activity quantification

The generation of ROS, such as hydroxyl radical OH − and superoxide anions can be detected using DCFH-DA^35^. A non-fluorescent cell membrane-permeable dye, DCFH-DA reacts with intracellular ROS, which emits fluorescent light. The fluorescence was measured using a standard protocol defined by^36^. After the addition of 2 µL of 100 M DCFH-DA to 2 ml of supernatant, the experimental samples of *P. roseola* were kept under dark conditions at room temperature for 30 min. The fluorescence of DCFH-DA was analyzed using an Eclipse fluorescence spectrophotometer (Cary, ModelG9800A, Agilent Technologies, USA) at excitation-emission wavelengths of 485 and 530 nm respectively.

The statistical analysis for the total protein content and the antioxidant assays (SOD, LPO, and ROS production) is expressed in the form of mean ± % Coefficient of variation (%CV) (n = 3) using Graph pad prism (GPP) software (Version 5.0). One-way analysis of Variance (ANOVA) was used to analyze the significant differences between the control and the treated groups. The significant differences among means (P ≤ 0.05) were tested using Dunnet’s test.

### Hatchability and Reproduction studies

The eggs were collected from the maintained stock culture of freshwater rotifers. They were stored in Eicosapentaenoic acid (EPA) medium. The eggs were used for a hatchability test. They were treated with PSNPs (50 and 100 nm) at different concentrations. The concentrations for the hatchability test were subsequently fixed based on the results obtained from the acute toxicity study carried out with adult rotifers. The LC_50_ values for 50 and 100 nm PSNPs were fixed as 4, 8, 12 and 16 mg/L. The time taken for hatching was recorded. Eggs that were not treated with PSNPs served as controls. A count of 100 eggs was taken in triplicates for each concentration and used during the experiments. The study was carried out for up to 48 h for both the PSNPs at the respective concentrations stated above. The (%) of hatched eggs at each time stage were carefully noted to compare with the control and the PSNPs-treated eggs.

For reproductive analysis, the method described by^37,38^ was followed with modifications. Newly hatched rotifers less than 2 h old (neonate stage- F_0_) were used for the experiment and were observed for 15 days (until the end of their lifecycle). A pair of male and female rotifers was added in a set of 10 replicates in a 24-well plate and exposed to different concentrations of PSNPs ranging from 4, 8, 12 and 16 mg/L for 50 and 100 nm PSNPs based on the LC_50_ values similar to the hatchability assay. Eggs that were not treated with PSNPs served as controls. Initially, reproduction was observed every 12 h. Neonates (F_1_) were collected every 12 h until the mortality of the adult (F_0_). The life cycle of F_0_ generation was studied by observing the time for egg brooding and total offspring produced until mortality of F_0_. The eggs collected from the adult F_0_ generation were chosen to study the emergence of the F_1_ generation. The results were observed under a phase contrast microscope (Leica microsystems) to observe the changes from the F_0_ to F_1_ generation every 12 h. In the control, F_0_ generation is produced up to F_4_ generation throughout the lifecycle. The eggs obtained from the rotifers were cultured in a sterilized EPA medium and were stored at 20℃ for future applications.

All the data were subjected to statistical analysis by one-way ANOVA and presented as mean ± S.D. (Significant differences) among means (P ≤ 0.05) were tested using Duncan’s multiple range test. Statistical data analysis of the results was done using GPP 5.0 software.

## Results

### Characterization of PSNPs

**Fig. 2:**
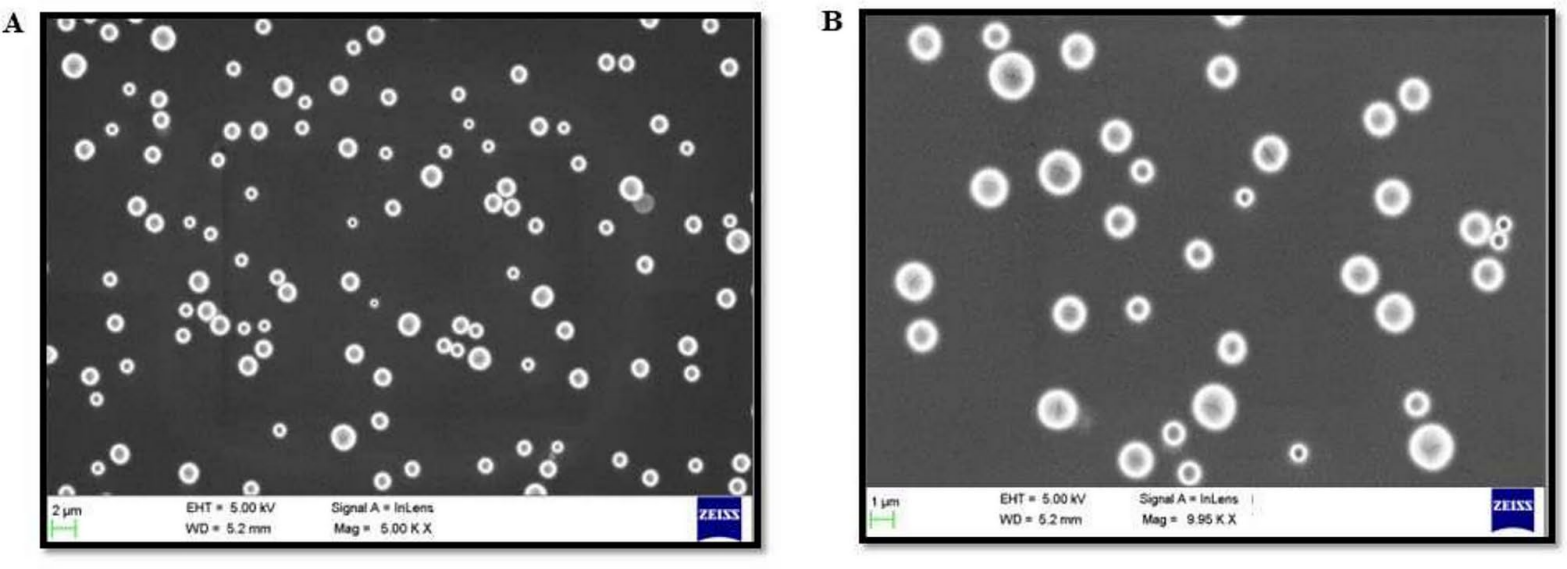
FE-SEM showing the spherical surface morphology of (**A**) 50 nm and (**B**) 100 nm PSNPs.

The surface morphology of the different sizes of PSNPs (50 and 100 nm) was characterized by (FE-SEM and NPs appeared spherical in shape as observed in Fig. 2A,B. The FESEM analysis revealed the size of PSNPs as 50 and 100 nm.

**Fig. 3:**
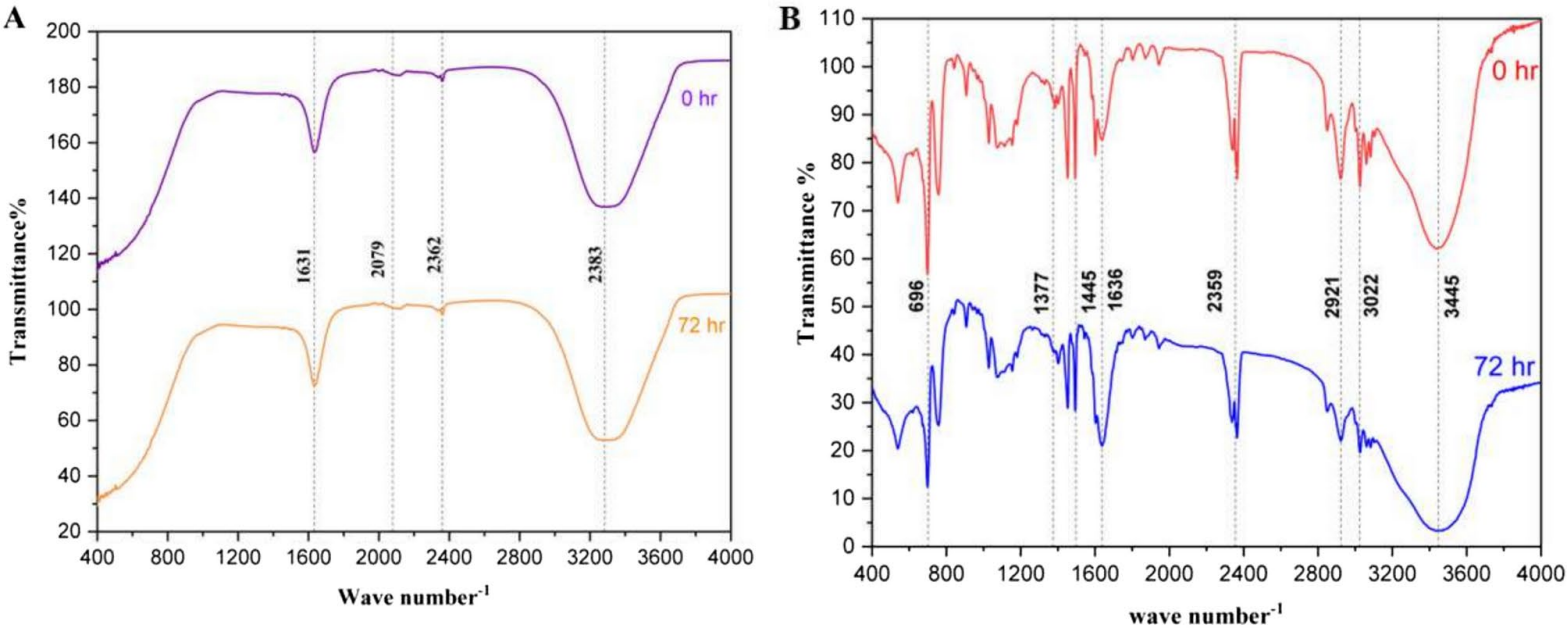
The FTIR spectral data of (**A**) 50 nm and (**B**) 100 nm PSNPs at 0 h and 72 h.

The FTIR spectral data of PSNPs (50 and 100 nm) at 0 and 72 h is shown in Fig. 3A,B. The given IR spectra report significant peaks and their respective functional groups. In the 50 nm PSNPs spectrum (Fig. 3A), a band at 1631 cm⁻^1^ indicates C = C stretching caused due to alkene/ aromatic bonds caused by PSs. The other band at 2079 cm⁻^1^ indicates alkynes C≡C and nitriles (C≡N). The peaks at 2362 and 2383 cm⁻^1^ indicate carbon dioxide (CO_2_) attributed to the adsorption of CO_2_ on the sample’s surface. In the 100 nm PSNPs spectrum (Fig. 3B), a peak at 696 cm^-1^ for C-H bending vibration for aromatic compounds was identified. The peaks at 1377 and 1636 cm^-1^ correspond to the methyl groups (CH_3_) and alkene/aromatic stretching (C = C) caused by PSs. A peak at 2359 cm^-1^ indicates CO_2_ presence which can be possibly attributed to the adsorption of CO_2_ on the sample’s surface. The peaks at 2921 and 3022 cm⁻^1^ are due to C-H stretching indicating the presence of aliphatic and aromatic hydrocarbons. The peak at 3445 cm⁻^1^ indicates O–H stretching for the presence of hydroxyl groups.

The sizes of the PSNPs were determined using DLS analysis. The results of 50 and 100 nm PSNPs at different concentrations from 24 to 72 h are tabulated in Table 1. The results revealed that the different concentrations of PSNPs aggregated within 72 h in both sizes (50 and 100 nm). When compared with the initial concentration of 20 mg/L, the aggregations of the PSNPs increased rapidly in the higher concentrations of 50 mg/L.

**Table 1:** DLS reports of different concentrations of 50 and 100 nm PSNPS analysed at 24 h to 72 h.

Different concentrations of the sample (mg/L)	50 nm PSNPs			100 nm PSNPs		
	24 h	48 h	72 h	24 h	48 h	72 h
10	59.3 ± 1.52	64.33 ± 2.08	78.33 ± 0.57	113 ± 1.73	121 ± 3	124 ± .3
20	63.0 ± 4	72.33 ± 4	83.33 ± 1.52	118 ± 3	124.3 ± 1.52	123 ± 4.58
30	66.33 ± 6.42	73.33 ± 5.13	81.33 ± 1.52	127.3 ± 1.15	132.3 ± 1.15	133.3 ± 1.15
40	63.0 ± 2	75.6 ± 2.51	85.0 ± 4	131.66 ± 6.65	137.33 ± 6.57	143.33 ± 6.65
50	66.66 ± 2.30	77.6 ± 2.30	85 ± 2	137.66 ± 6.35	139.33 ± 6.57	143.13 ± 6.35

### Acute toxicity assessment of the PSNPs in *Philodina roseola*

**Fig. 4:**
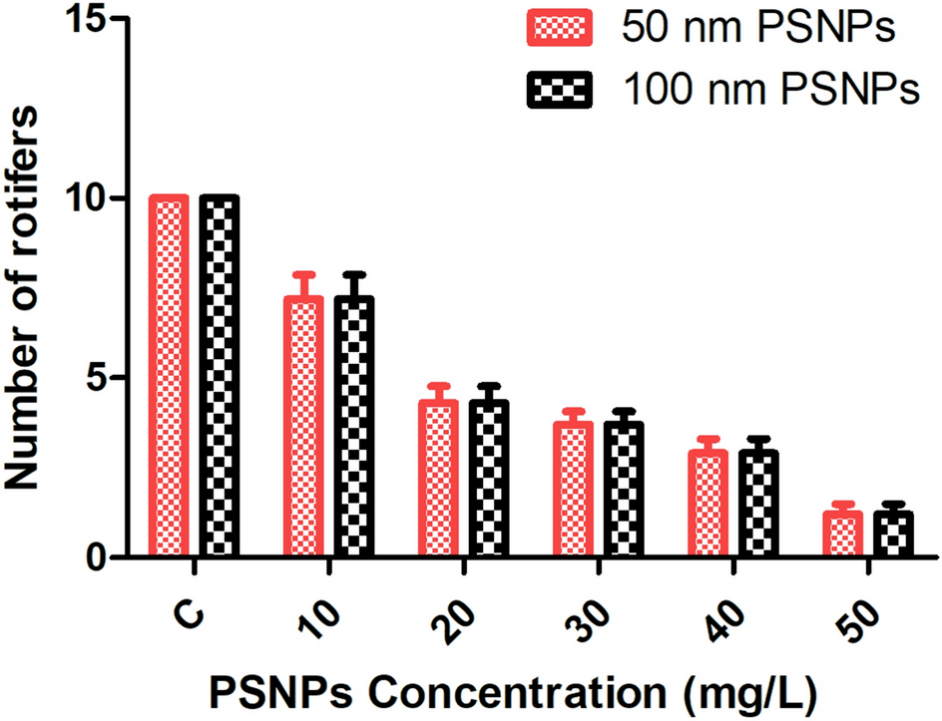
Graphs representing the decrease in number of rotifers (observed mortality) as the concentration increases at 48 h that were exposed to 50 and 100 nm PSNPs.

**Fig. 5:**
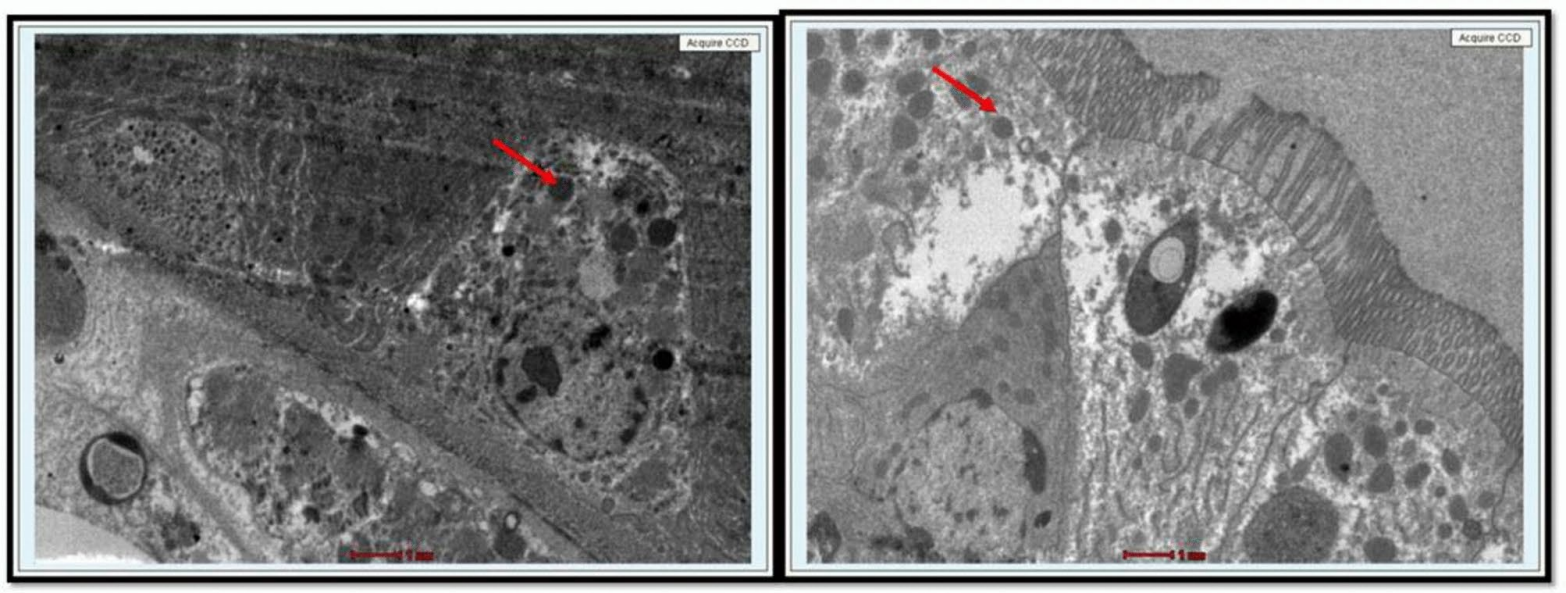
TEM showing the damage caused by the ingested PSNPs in rotifers which were treated above the range of 20 mg/L with 50 nm PSNPs.

**Fig. 6:**
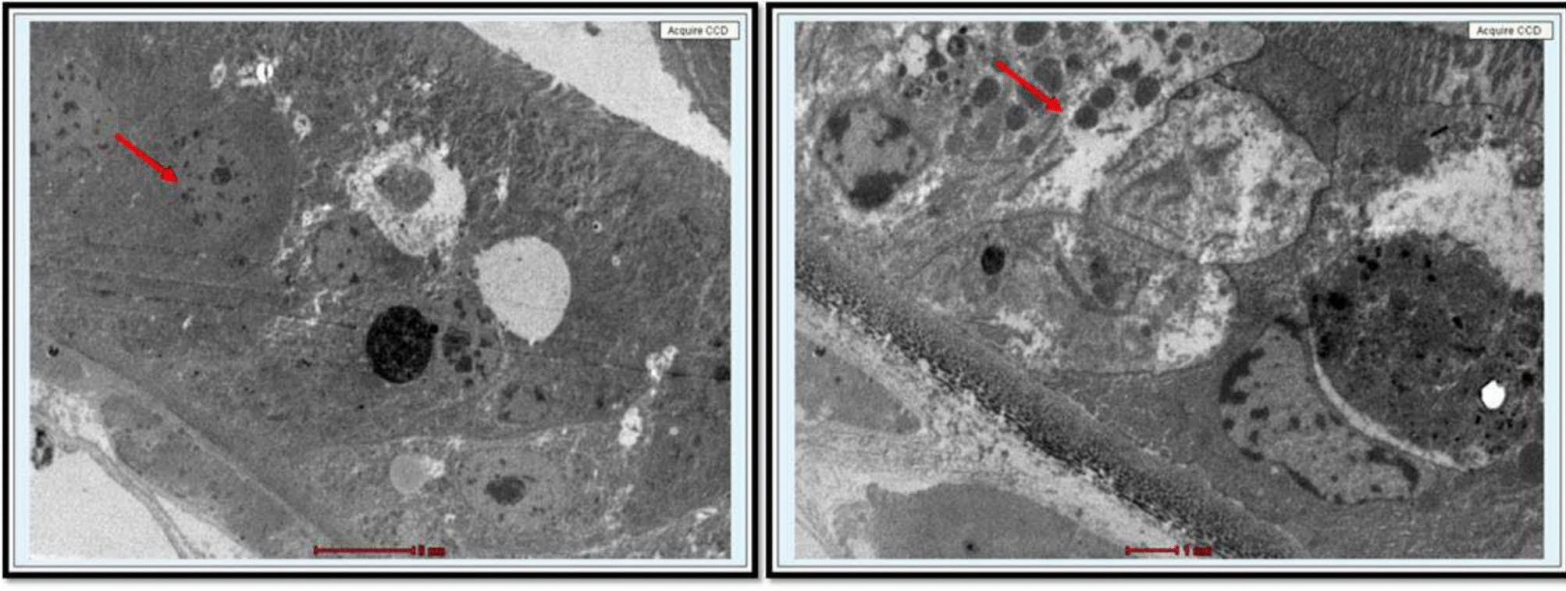
TEM showing the damage caused by the ingested PSNPs in rotifers which were treated above the range of 20 mg/L with 100 nm PSNPs.

The rotifer*, P. roseola*, was treated with PSNPs to study its toxicity, biochemical parameters, hatchability of eggs, and survival of adults. The acute toxicity test with rotifers was chosen because of its robustness among the standardized methods used for ecotoxicological studies^39^. Adult rotifers were studied for toxicity to PSNPs (50 and 100 nm) for a period of 72 h. When treated with 100 nm PSNPs, it was observed that the adult rotifers showed 29% mortality at 24 h, 49% mortality at 48 h, and 98% mortality at 72 h. The LC_50_ value for 100 nm PSNPs was found to be 22.94 mg/L at 48 h. When treated with 50 nm PSNPs, 38% mortality at 24 h, 53% mortality at 48 h, and 95% mortality at 72 h was observed in adult rotifers. Lethal concentration (LC_50_) values were calculated for adult rotifers treated with 50 and 100 nm PSNPs. The LC_50_ value for the 50 nm PSNPs was observed as 16 mg/L at 48 h**. **Figure 4 depicts the observed mortality at 48 h in rotifers that were exposed to 50 and 100 nm PSNPs. The probit analysis data used to identify the LC_50_ value is included in the supplementary file Fig. S2. When compared to the control, it can be clearly observed that at the highest concentrations, the damage to the rotifers was higher due to the ingestion of PSNPs in them. TEM was performed to examine the presence of PSNPs in the damaged internal organs of rotifers. The results revealed that PSNPs were ingested and caused damage in rotifers which were treated above the range of 20 mg/L of 50 and 100 nm PSNPs as observed in Figs. 5 and 6**.** In Figs. 5 and 6, the presence of PSNPs in the animals was observed. The observed damage in the rotifers is displayed in Fig. S3.

#### Biochemical parameters evaluation assay

**Fig. 7:**
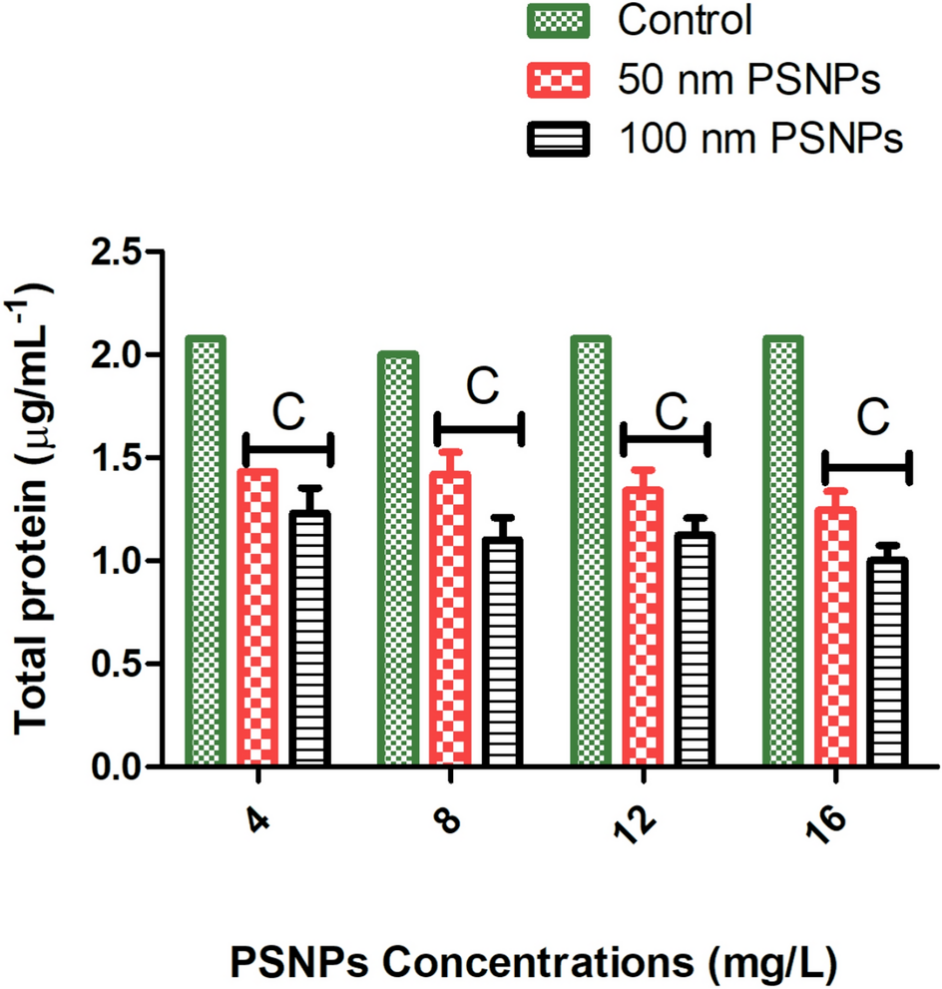
Graph showing the increase in total protein content with increasing concentrations when compared with the control and treated groups of 50 and 100 nm PSNPs (Expressed in the form of Mean ± %CV; n = 3; C indicates most significant (p < 0.001)).

**Fig. 8:**
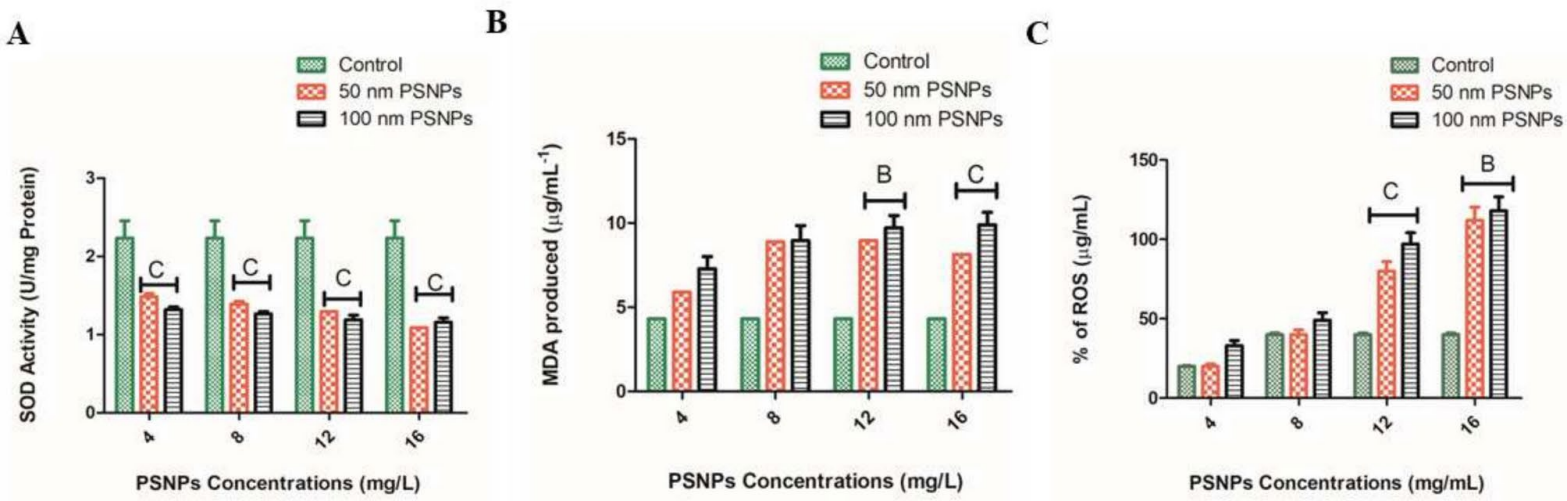
Graphs showing the antioxidant levels (**A**) SOD levels (**B**) LPO activity (MDA produced) (**C**) ROS levels showing the significant difference between control and the treated groups treated with 50 and 100 nm PSNPs (Expressed in the form of Mean ± %CV; n = 3; B indicates (p < 0.01) more significant; C indicates (p < 0.001) most significant).

Biochemical studies on zooplankton are an important approach to understand their metabolism, nutritive value, and role in the aquatic food chain, as reported by^40^. Based on the LC_50_ values at 48 h, the concentrations were fixed accordingly for the assays. There was an exponential reduction of protein content in rotifers treated with increasing concentrations of 50 nm PSNPs. For 100 nm, the level of protein decreased as the concentration of PSNPs increased compared to the control. In comparison to the 100 nm PSNPs, 50 nm PSNPs showed a significant decrease in protein content. However, the protein content of control organisms and the lowest concentration of the 100 nm PSNPs (4 mg/L) remained the same. The concentrations of protein in 50 nm PSNPs decreased from 1.432 ± 0 (4 mg/L) to 1.244 ± 3 (16 mg/L). Whereas in 100 nm PSNPs the protein concentration went from 1.230 ± 4 (4 mg/L) to 1 ± 3 (16 mg/L) (Fig. 7). For 50 nm PSNPs treated organisms, the SOD antioxidant activity decreases with the increasing concentration of the PSNPs. Whereas for 100 nm PSNPs treated organisms, the SOD activity decreases with increasing concentration. However, the SOD values of the control were found to be increased when compared to both PSNPs treated organisms (p < 0.05). Similar to the protein level, SOD activity decreased from 1.486 ± 1 (4 mg/L) to 1.089 ± 0 (16 mg/L) in 50 nm PSNPs and 1.320 ± 1 (4 mg/L) to 1.156 ± 2 (16 mg/L) in 100 nm PSNPs (Fig. 8A). The variations in antioxidant assays are due to the oxidative stress caused by the NPs towards the test organisms. Using the biochemical technique, xanthine-xanthine oxidase is used for generating O_2_^•−^ and reduction of NBT is used generally as an indicator for O_2_^•−^ production. The SOD will engage with NBT for O_2_^•−^ during the process^41^. The presence of free radicals produces the LPO in a particular organism. MDA is one of the end products formed from the peroxidation of polyunsaturated fatty acids at the cellular level. Whereas the significant increase in free radicals is responsible for the overproduction of MDA^42^. The mutagenic secondary product MDA^43^ is produced due to the oxidative stress in the test animals and was analyzed through LPO assay. It is widely used as a convenient biomarker to assess LPO activity. The LPO range of controls was comparatively low when compared with the PSNPs-treated organisms. The lipid peroxidation assay revealed that the MDA values increased with the increasing concentration for both the treated rotifers (50 nm and 100 nm). The MDA production activity for 50 nm PSNPs increased from 5.89 ± 0 (4 mg/L) to 8.13 ± 0 (16 mg/L). Whereas in 100 nm PSNPs, the MDA production increased from 7.29 ± 4 (4 mg/L) to 9.91 ± 3 (16 mg/L) (Fig. 8B). ROS results revealed that the oxidative stress causes the release of free radicals depending on size and concentrations of PSNPs in rotifers. An increase in the concentration of PSNPs resulted in the enzymes reacting to release a low percentage of radicals at 100 and 50 nm. The ROS activity increased with the increasing concentrations. The ROS levels increased from 20 ± 3 (4 mg/L) to 112 ± 3 (16 mg/L) in 50 nm PSNPs and 33 ± 4 (4 mg/L) to 118 ± 3 (16 mg/L) in 100 nm PSNPs (Fig. 8C). The MDA production (LPO) and ROS levels increased with increase in concentration for both PSNPs interacted with the rotifers.

### Hatchability studies

**Fig. 9:**
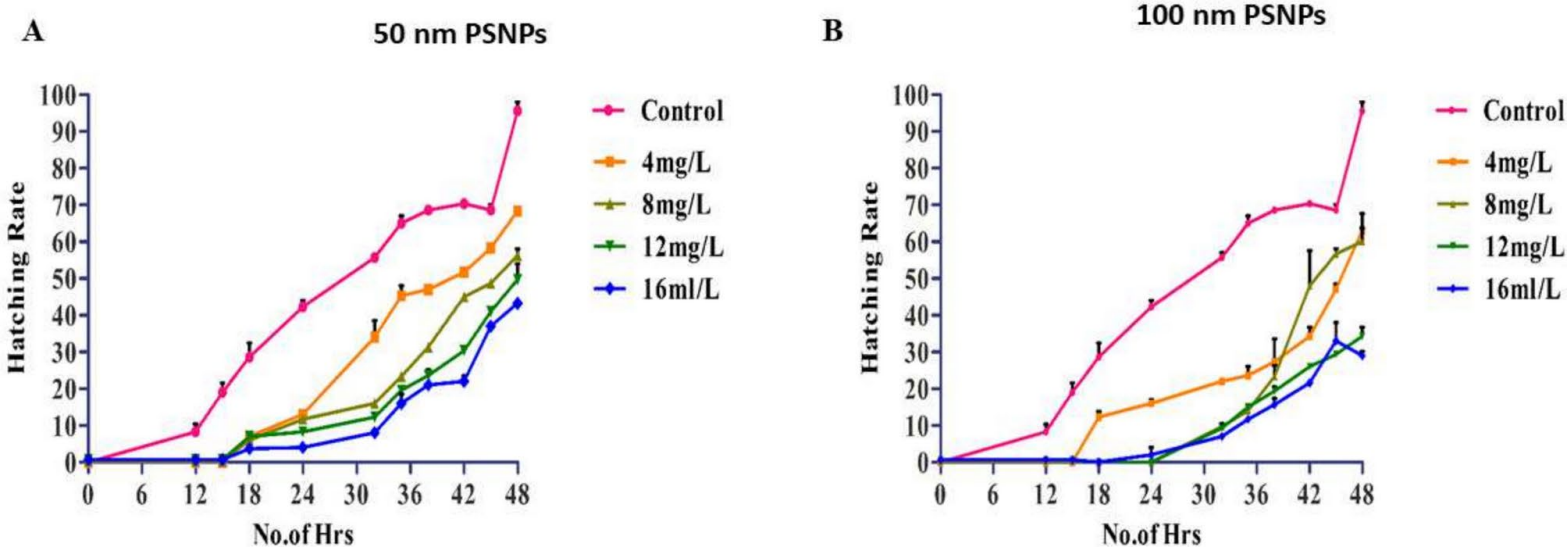
The graphs representing the hatchability rate of the eggs with (**A**) 50 nm PSNPs and (**B**) 100 nm PSNPs at different concentrations (Expressed in the form of Mean ± S.D; n = 3).

In the control eggs, almost 90% of eggs hatched into neonates within 48 h, and 100% hatchability of the eggs was observed at 120 h. The results revealed that there was a delay in hatching when the eggs were treated with 50 and 100 nm PSNPs. For 50 nm PSNPs concentrations, 52% of the eggs hatched after 48 h in the lowest concentration (4 mg/L) and only 22% hatchability in the eggs was observed in the highest concentration (16 mg/L) at 48 h. The graph representing the hatchability of the eggs with 50 nm PSNPs at different concentrations is depicted in Fig. 9A. It is obvious from the graph that the increase in concentration leads to a decrease in the hatchability of the eggs. Similarly, 100 nm PSNPs show a decreased hatchability rate with an increase in the concentration of PSNPs as observed in Fig. 9B. For 100 nm PSNPs, 68% hatchability in the eggs was observed in the lowest concentration (5 mg/L) and 35% of the eggs hatched at the highest concentration (20 mg/L). The obtained results clearly indicate the interaction of PSNPs with the rotifers affects their lifecycle by delaying the hatching process.

### Reproduction studies

**Fig. 10:**
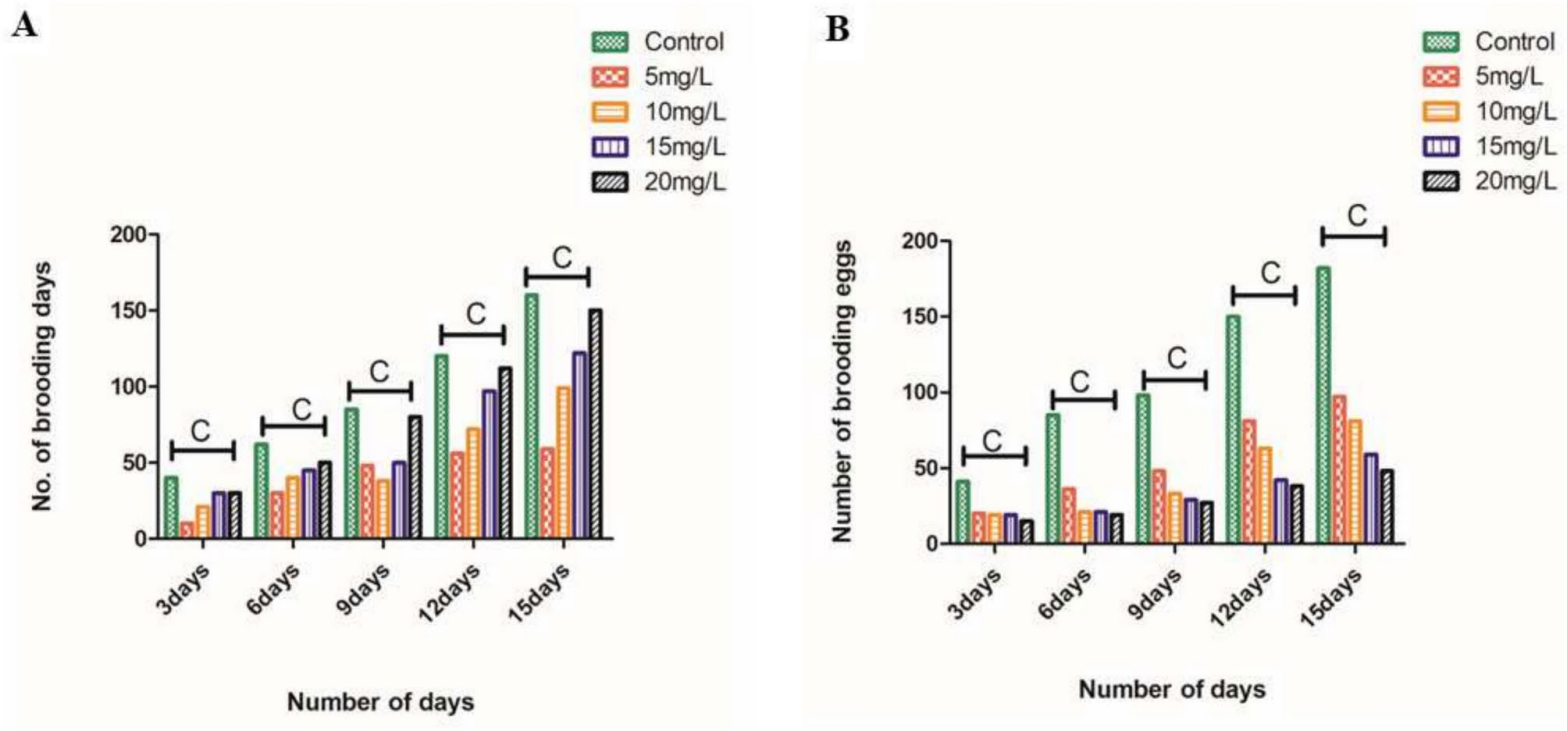
The graphs representing the brooding eggs (hatching eggs) at F_1_ generation when exposed to (**A**) 50 nm PSNPs and (**B**) 100 nm PSNPs at different concentrations in various time periods (Expressed in the form of Mean ± S.D; n = 3; C indicates(p < 0.001) most significant).

Reproduction studies were carried out to determine the number of generations that the rotifers were able to produce during their lifecycle (15 days). It was observed that the control animals were able to reproduce neonates up to F_4_ generations (F_0_-F_4_), whereas the animals treated with 50 and 100 nm PSNPs were able to reproduce only up to F_1_ generation during its lifecycle. The brooding eggs produced during its lifecycle of 15 days for 50 and 100 nm PSNPs in F_1_ generation are shown in Fig. 10A,B. The results revealed that as the concentration of PSNPs increased, toxicity also increased in the eggs and adults. It was observed that neonate and adult rotifers ingested PSNPs. The results were found to be statistically significant with ANOVA studies.

## Discussion

The current study focuses on the toxic effect of PSNPs on *P. roseola*. The chemical characterization of PSNPs gives an overview of the structure and the interaction of the PSNPs with the fresh water. The PSNPs used in the study were observed as spherical particles by FESEM analysis. In the FTIR data, it can be observed that the transmittance of the peaks decreased between 0 h (blue spectrum) and 72 h (red spectrum) indicating the possibilities for degradation of a specific functional group or low concentration of the sample over the specified time period. The lowering of peak intensity in the samples reveals the structural alteration caused by to modification of chemical composition. Reports that focus on the impacts of NPs mostly use the PSNPs of the sizes 50 and 100 nm. The collective literature survey carried out by^44^ with a special focus on bioaccumulation and toxicity of PSNPs on various marine and terrestrial organisms shows multiple reports of the interaction of PSNPs in the specified size used in the current study.

In the current study, the PSNPs of both sizes caused toxicity to the test animals. LC_50_ is the concentration required to kill 50% of a large population of the test species. This is achieved by determining the response of an organism to different concentrations of chemicals and then comparing the concentrations at which a response occurs^19,45^). The present study shows the toxicity and lethal effects of PSNPs (50 and 100 nm) implicating the rotifers upon exposure. The LC_50_ values indicate the decrease in the number of rotifers as the concentration of the PSNPs increases. The range below the LC_50_ was used to study the biochemical and antioxidant parameters, hatchability, and reproducibility tests.

The biochemical studies carried out on the rotifers caused significant differences in their total protein and antioxidant levels. For both sizes of the PSNPs, the total protein and the SOD levels decreased with the increasing concentration of the PSNPS. Contrarily, the LPO activity and ROS activity increased with the increasing concentration of the PSNPs.

*B. plicatilis* rotifers exposed to various concentrations of NPs and ZnO showed a delay in their sexual maturation time and a significant decrease in the total offspring count with increasing concentrations of NPs. The overall survival rate, development, and reproduction rate of the rotifers were greatly affected^46^. The effect of exposure of the same rotifer to different sizes of MNPs was studied. Molecular analysis was carried out to identify the underlying functional transcriptomic responses caused by the plastics. In the size-dependent exposure study of NPs and *B. plicatilis*, NPs were reported to cause more toxicity than the MPs. The study states that only NPs generated oxidative stress in the organism. Further, retarded population growth and reproduction levels were greatly recorded in NPs more than MPs. Further, transcriptomic analysis revealed the metabolic deficiency caused by MNPs exposure^47^. Multigenerational resilience of *B. plicatilis* exposed to NPs in high temperature and salinity conditions was reported in a study. Stress elevation (increased ROS levels and other antioxidant enzymes) along with similar toxic effects observed earlier were identified in the study. These conditions further decreased the fatty acid profiles of the organisms and caused a significant decrease in their overall lifespan. Overall, the recovery from higher temperatures was more rapid than the other conditions tested^48^.

Similar exposure studies of MNPs on *B. plicatilis* under hypoxia conditions, revealed the overproduction of the ROS and reduction in the fertility of the organisms. The pathways of the hypoxia-inducible factor and the circadian clock genes of the test organisms were greatly upregulated under these conditions. These changes were dependent on the size of the MNPs exposed. Further, even after several generations of exposure, the reduction in transcriptomic resilience and the reproductive rate remained the same. In contrast, the ROS production returned to the basal range providing insights on the toxic effects of MNPs after multiple generations^49^. *Daphnia magna,* a micro-level aquatic organism was studied with similar conditions. The ROS levels and the antioxidant enzyme were highly upregulated because of its exposure to NPs under hypoxia conditions. As a result of single-stressor exposure, growth impairments and reproductive systems were significantly observed^50^. All these studies highlight that exposure to NPs has a significant effect on the organism affecting its lifecycle by altering its metabolism at the cellular level.

The MNPs can easily penetrate into the aquatic food chain (lower to higher level organisms) through sources such as plankton by the transport, ingestion, and accumulation process. These organisms disrupt the overall metabolism and reproduction cycles by aggregating into the cells and tissues of aquatic organisms^51^. A study by^52^ studied the food chain transfer effects of MNPs from rotifers to *B. plicatilis* to marine medaka (*Oryzias melastigma*). In the study, the rotifers were evaluated to analyze the transfer of PMNPs and toxicity of PS-NPs on marine medaka over continuous food chain exposure. This study is an example of the transfer of toxicity from rotifers to higher-order organisms. As observed from the FTIR and DLS results, the aggregation of these PSNPs in the aquatic system will ultimately affect the hatchability of the eggs and the animals in general. Similarly, in the eggs exposed to 100 and 50 nm PSNPs for 24 and 48 h, there was damage to the cyst of rotifers at different test concentrations. These findings were further supported by the reproductive studies based on the generations produced by these test animals. In our study, when *P. roseola* was fed with PS, toxicity was higher at higher concentrations. All these reports confirm the interference of PSNPs with the rotifers by showing toxicity and reducing the hatchability of the eggs.

Additionally, studies have shown that plastics larger than 100 nm accumulate in the gut until excretion, whereas smaller plastics (50 nm) are taken up by cells and transferred from mothers to the eggs, resulting in reduced reproduction and feeding rates^53^. Reference^16^ studied the toxicity of PSNPs to marine rotifers (*B. plicatilis*) when exposed to 0, 20, 200, and 2000 μg/L of 70 nm PSNPs for two generations (F_0_ − F_1_), followed by a two-generation (F_2_ − F_3_) culture in clean seawater, to determine its impact on life-history traits. The results showed that NPs were ingested by rotifers within 10 min and reached a maximum level after 12 h of exposure. NPs were also observed in the feces of F_0_ and F_1_ generation rotifers and on the surface of F_1_ generation eggs. Additionally, MNPss have been found to have significant size-dependent adverse effects on growth, reproduction, ROS production, and antioxidant responses in rotifers. Reference^53^ reported that only the smaller sized MPs (0.07 μm) at higher concentrations had a strong negative effect on life-history traits of *P. globosa*, Overall performance of the rotifer was weakened by 0.07 μm MPs at 5 μg mL^−1^ or above. The possible toxicity of the 100 nm NPs is due to the accumulation and aggregation of the plastics inside the organism. The 50 nm NPs can be easily assimilated into the cell, whereas the 100 nm NPs can be possibly aggregated in the tissues and accumulated over a time period causing more toxic effects than the smaller size. As per the results of^54^, *D. magna* rapidly ingested the MNPs even at a low-dose exposure, with plastics of larger size and positive charge being ingested in higher amounts but egested in lower amounts. This study is an example of the accumulation of larger size plastics inside the organism causing toxic effects. They reported that at higher concentrations of NPs, eggs were completely damaged. These reports corroborate the present study results with reduced reproduction rates caused by these PSNPs. It can be concluded that NPs can be more damaging to rotifers than larger-sized MPs. NPs can damage cell membranes in microscopic marine organisms, such as rotifers. MPs have become pervasive environmental pollutants in both freshwater and marine ecosystems.

The present study revealed that rotifers treated with PSNPs of 100 and 50 nm showed toxicity at higher concentrations. The increasing concentration of PSNPs caused lethal effects to the rotifers altering their protein and antioxidant parameters. PSNPs reduced the hatching rate of the rotifer eggs when compared to the control. Further, the rotifer treated with PSNPs reduced the reproductive stage of the rotifer by growing to F_1_ generation only. This proves that the presence of MNPs in the aquatic environment could hinder their growth. It can be concluded that plastic pollution (specifically nanoplastics) in the aquatic environment can be toxic to the lower level of organisms (zooplankton) which are in the food chain and can significantly influence the aquatic food chain. This study is an initial attempt to unravel the toxic effect of PSNPs on the rotifer (*P. roseola*).

## Supplementary Information

Supplementary Information.

## Data Availability

The raw datasets analyzed during the study is available from the corresponding author on reasonable request.
