# Supplementary material for: Effect of polystyrene nanoplastics on its toxicity and reproduction in Philodina roseola
Source: Sci Rep. 2025 Apr 23;15:14206. doi: 10.1038/s41598-025-98637-1 (PMC12019363; doi:10.1038/s41598-025-98637-1)
Supplement: Supplementary file 1 — Supplementary Information. [file 41598_2025_98637_MOESM1_ESM.docx]

**Supplementary file**

**Fig.S1.** The image of *P. roseola* used for interacting with PSNPs.

**
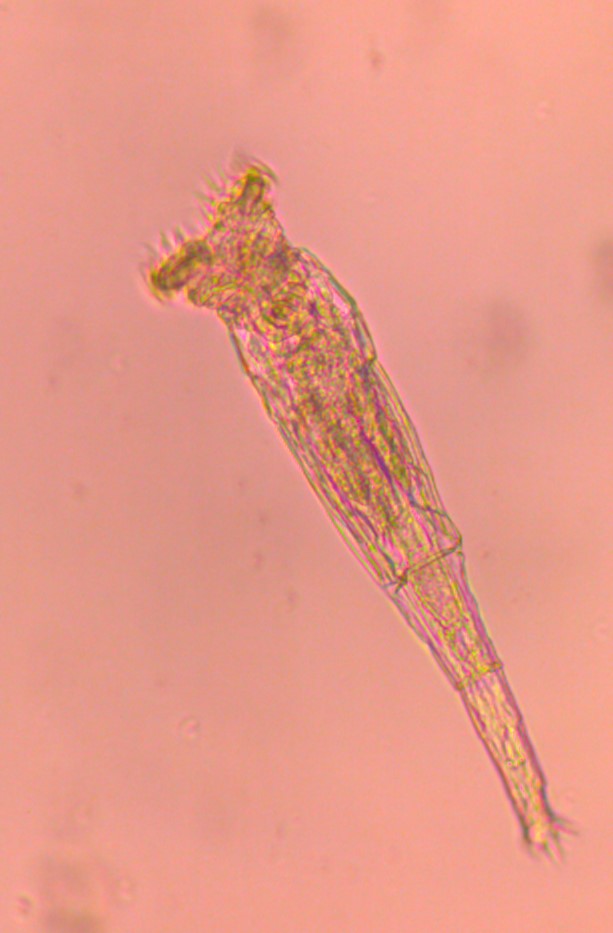
**

**Fig.S2.** The probit analysis data used to identify the LC_50_ value in A) 50 and B) 100 nm PSNPs treated rotifers


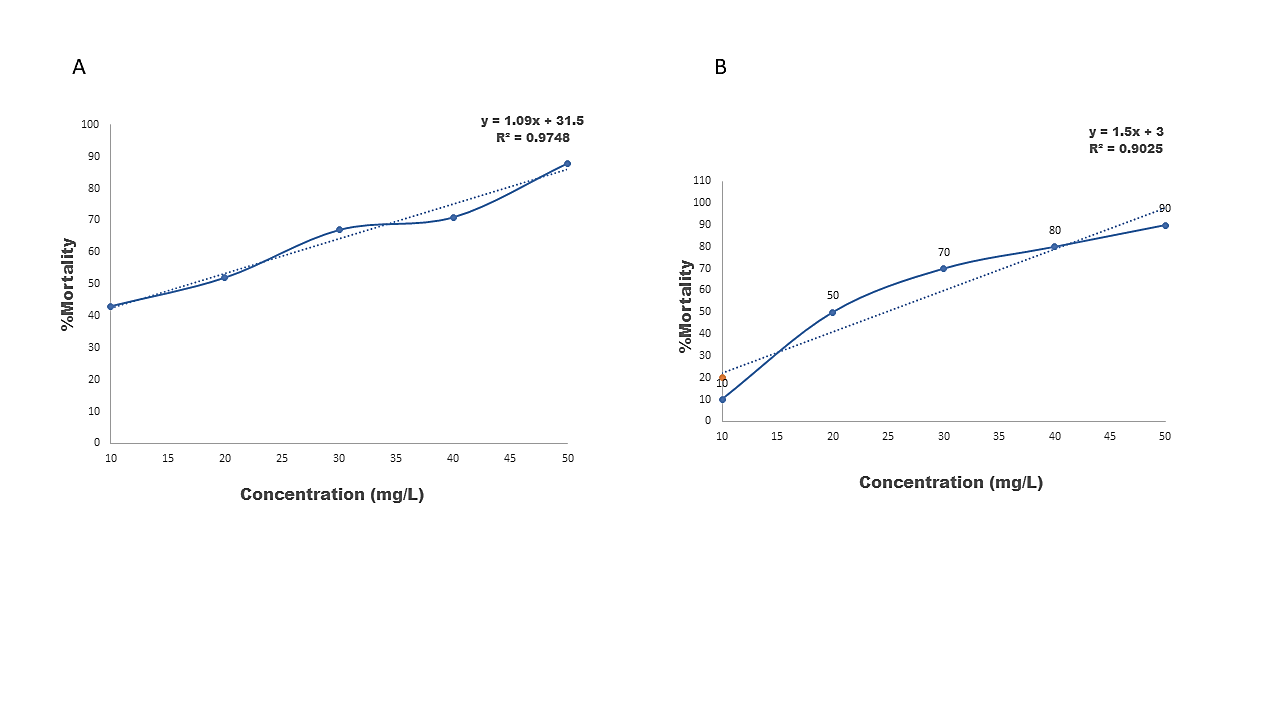


**Fig.S3** A) Control; B and C) 100 and 50nm of PSNPs (20ppm); D and E) Effects F1 and F2 adults to laid eggs in 100 and 50nm PSNPs. F) F_1_ generation showing decreased number of eggs to lay within 2 days(48hrs).

**
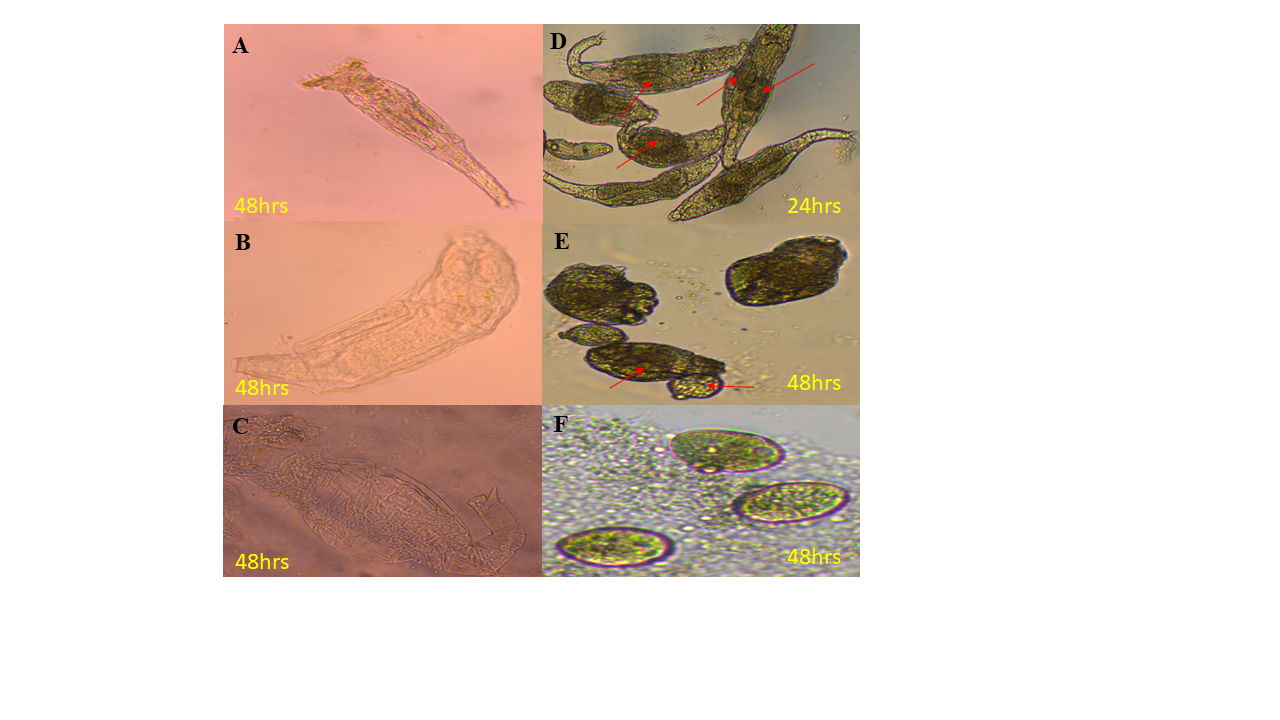
**
